# The Orbiting Carbon Observatory (OCO-2) tracks 2–3 peta-gram increase in carbon release to the atmosphere during the 2014–2016 El Niño

**DOI:** 10.1038/s41598-017-13459-0

**Published:** 2017-10-19

**Authors:** Prabir K. Patra, David Crisp, Johannes W. Kaiser, Debra Wunch, Tazu Saeki, Kazuhito Ichii, Takashi Sekiya, Paul O. Wennberg, Dietrich G. Feist, David F. Pollard, David W. T. Griffith, Voltaire A. Velazco, M. De Maziere, Mahesh K. Sha, Coleen Roehl, Abhishek Chatterjee, Kentaro Ishijima

**Affiliations:** 1 RCGC/IACE, Japan Agency for Marine-Earth Science and Technology (JAMSTEC), Yokohama, 236-0001 Japan; 2 Jet Propulsion Laboratory, California Institute of Technology, Pasadena, CA USA; 3 Max Planck Institute for Chemistry, Mainz, Germany; 4 Department of Physics, University of Toronto, Toronto, Canada; 5 Project Team for HPC Advanced Predictions utilizing Big Data, JAMSTEC, Yokohama, 236001 Japan; 6 California Institute of Technology, Pasadena, CA USA; 7 Max Planck Institute for Biogeochemistry, Jena, Germany; 8 National Institute of Water and Atmospheric Research Ltd (NIWA), Lauder, New Zealand; 9 School of Chemistry, University of Wollongong, NSW, 2522 Australia; 10 Royal Belgian Institute for Space Aeronomy, Brussels, Belgium; 11 Universities Space Research Association, Columbia, MD, 21046 USA; 12 NASA Global Modeling and Assimilation Office, Goddard Space Flight Center, Greenbelt, MD, 20771 USA; 13 Present Address: Center for Global Environmental Research, National Institute for Environmental Studies, Tsukuba, 305-8506 Japan; 14 Present Address: Center for Environmental Remote Sensing (CEReS), Chiba University, Chiba, Japan

## Abstract

The powerful El Niño event of 2015–2016 – the third most intense since the 1950s – has exerted a large impact on the Earth’s natural climate system. The column-averaged CO_2_ dry-air mole fraction (XCO_2_) observations from satellites and ground-based networks are analyzed together with *in situ* observations for the period of September 2014 to October 2016. From the differences between satellite (OCO-2) observations and simulations using an atmospheric chemistry-transport model, we estimate that, relative to the mean annual fluxes for 2014, the most recent El Niño has contributed to an excess CO_2_ emission from the Earth’s surface (land + ocean) to the atmosphere in the range of 2.4 ± 0.2 PgC (1 Pg = 10^15^ g) over the period of July 2015 to June 2016. The excess CO_2_ flux is resulted primarily from reduction in vegetation uptake due to drought, and to a lesser degree from increased biomass burning. It is about the half of the CO_2_ flux anomaly (range: 4.4–6.7 PgC) estimated for the 1997/1998 El Niño. The annual total sink is estimated to be 3.9 ± 0.2 PgC for the assumed fossil fuel emission of 10.1 PgC. The major uncertainty in attribution arise from error in anthropogenic emission trends, satellite data and atmospheric transport.

## Introduction

Uncertainties in estimates of regional sources (+ve flux) and sinks (−ve flux) of CO_2_ and other greenhouse gases, derived from direct inventory methods or inferred from atmospheric observations, have hindered the development of effective policy for reduction of emissions from anthropogenic activity^1^. The large uncertainties obscure the relative roles of management approaches for terrestrial biospheric fluxes and the energy intensity of the industrial activities. For example, the sources and sinks of CO_2_ by the tropical land biosphere has remained uncertain^2^ and the CO_2_ emissions from industries in China are frequently revised by the state and international research communities^3^. While the inventory method suffers from a lack in completeness and transparency, the atmospheric constraint has hitherto been compromised by both the sparseness of observational network, and uncertainties in models employed for regional CO_2_ flux calculations^4^.

To improve the time and spatial coverage of the atmospheric CO_2_ measurements, NASA launched the OCO-2 satellite in July 2014^5^. Since early September of 2014, OCO-2 has been routinely returning almost one million soundings each day over the sunlit hemisphere. While clouds and large aerosols abundances preclude full-column measurements of CO_2_ from most of these soundings, more than 10% (~100,000 soundings/day) yield estimates of the column-averaged dry air mole fraction, XCO_2_. The OCO-2 XCO_2_ retrievals, after bias correction, agree well globally with the TCCON for nadir, glint, and target observations, with median differences less than 0.5 parts per million (ppm) and root-mean-square differences typically below 1.5 ppm^6^. If regional scale biases are controlled to similar levels, these data can provide the precision and accuracy needed to characterize CO_2_ sources and sinks^7^.

The other factor that affects estimates of CO_2_ fluxes from XCO_2_ measurements is the biases in the inverse methods using chemistry-transport models (CTMs). The role of such bias has been illustrated using the XCO_2_ observations from the first dedicated Greenhouse Gases Observing Satellite “IBUKI” (GOSAT), which was launched on 23 January 2009 by the Japan Aerospace Exploration Agency (JAXA)^8^. Using multiple flux inversions of *in situ* and satellite CO_2_ data, Howeling *et al*. find that the model-model flux differences quickly increase to >100% of the annual flux on the scale of the subcontinental regions^9^. It is generally understood that the differences in inversion-derived CO_2_ fluxes are caused by a variety of the underlying modeling components in the inversion systems, not the CTMs alone^4,9^. The modeling components include a priori flux and uncertainty assumptions, screening and treatment of observational data, and uncertainties in transport models^4^.

The efficiency of the terrestrial ecosystem at absorbing atmospheric carbon dioxide (CO_2_) depends on the availability of sunlight, soil moisture (fed by precipitation), and air temperature^10,11^. Thus droughts and high temperatures associated with El Niño reduce the ability of the terrestrial ecosystem to assimilate carbon while additional release by frequent occurrence of fires further reduces the uptake of carbon by the terrestrial biosphere^12–16^. The pyrogenic carbon flux of Indonesia during 2015 has been estimated with bottom-up methods from fire observations by the MODIS satellite instruments and with top-down, i.e. inversion, methods from atmospheric CO observations by the MOPITT satellite instrument. The bottom-up methods yield values of 340 TgC^17^, 380 TgC^16,18^ and 408 TgC^19^ for all of 2015, and of 250 TgC^17^ and 320 TgC^19^ for September-October 2015. The two CO inversions yield higher estimates (501 ± 170 TgC^20^ for all of 2015 and 227 ± 66 TgC^21^ for September-October 2015). The study regions are all dominated by the Indonesian fires despite varying in their exact definitions (“Tropical Asia”, “Maritime Southeast Asia” etc.). The range of estimates provides some measure of the considerable uncertainty in our knowledge of the pyrogenic carbon flux. However, each of these anomalies is smaller than those estimated for the 1997/1998 El Niño event for Southeast Asia (~1 PgC)^14,16^.

In addition to the relatively large uncertainties, the above-mentioned carbon flux estimates are limited only to the emission mechanism of biomass burning. CO_2_ observations, on the other hand, have the advantage of being more directly linked to the net carbon flux to the atmosphere, i.e., they are not limited to a specific emission mechanism like biomass burning.

Although the equatorial east Pacific Ocean experiences weaker ventilation of deep-water CO_2_ during an El Niño, thus a negative CO_2_ flux anomaly^22^, but the effect of the ocean component on global total CO_2_ flux anomaly is not clear^15,23^. For simplicity of this work, no attempt is made to partition land and ocean fluxes.

Here, we analyze early OCO-2 observations of XCO_2_ to quantify the impact of the powerful El Niño event^24^ on large scale CO_2_ flux anomalies. A record CO_2_ rise is predicted for 2016, sufficient to keep the atmospheric level above 400 ppm at Mauna Loa, Hawaii^25^ for the foreseeable future. The OCO-2 observations along with CTM simulations are used to make an impact assessment of the ongoing El Niño event on the terrestrial carbon cycle. We estimated monthly CO_2_ flux corrections from the differences in OCO-2 measurements and transport model simulations. Comparisons with *in situ*, ground-based remote sensing and GOSAT observations provide a test of the robustness of the estimated carbon exchange based on the OCO-2 observations.

## Results

### Model-observation comparison

**Figure 1:**
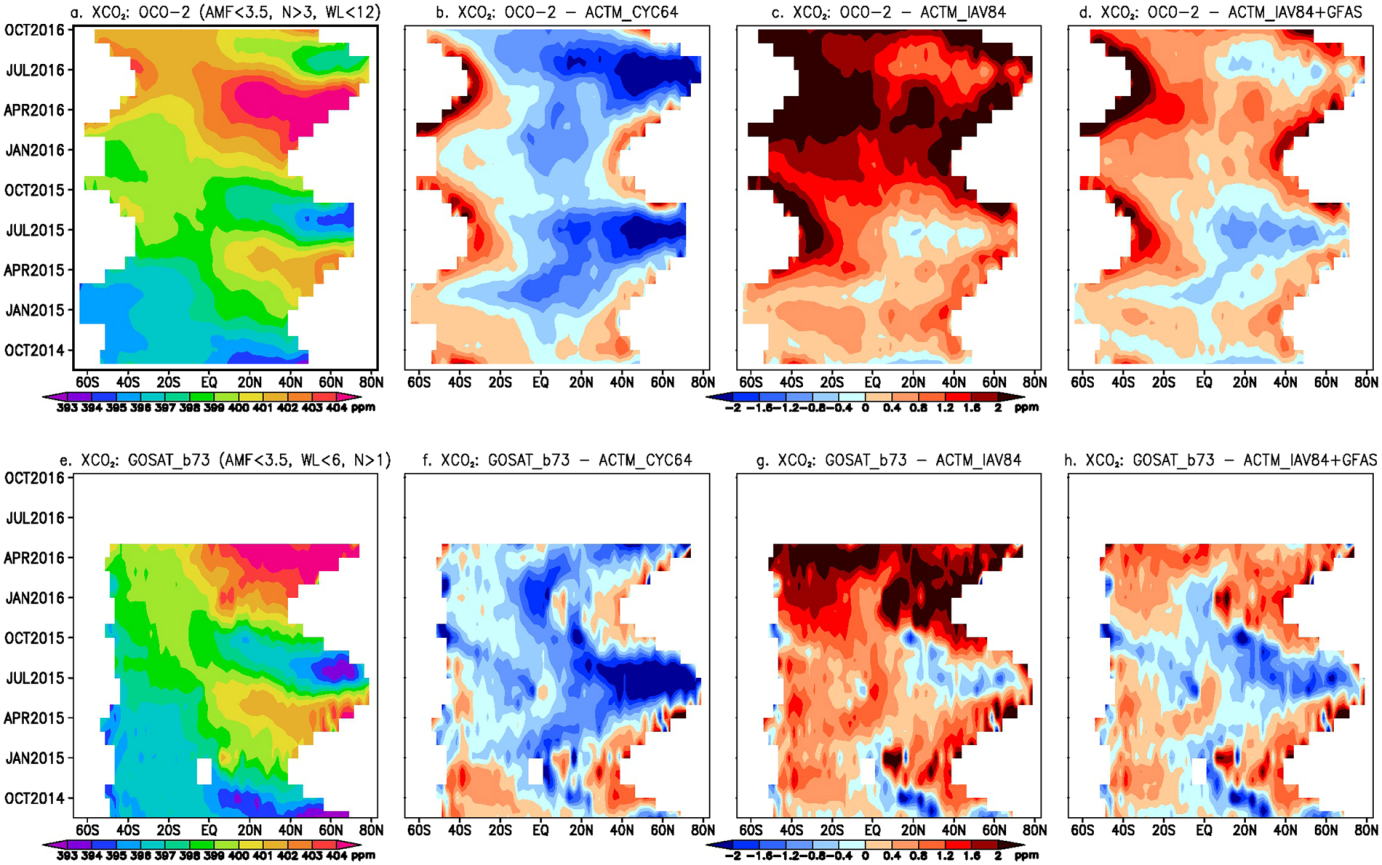
Time evolution of XCO_2_ from satellites and model. Latitude-time distribution of XCO_2_ (in ppm) measured from OCO-2 (**a**) and GOSAT (**e**), and their differences with 3 cases of ACTM simulations (**b**–**d** and **f**–**h**, respectively) for the period of OCO-2 operation, from 07 September 2014 to 31 October 2016 (GOSAT ACOS b7.3 are available until 31 May 2016). Note the striking similarities between OCO-2 and GOSAT measurements and ACTM_IAV84 + GFAS simulation case, particularly over the tropics. Further detailed comparisons of GOSAT and ACTM, with separation for soundings over land and water surfaces suggests the positive model biases in the high latitude regions arise mainly over the ocean surface. Similar plots cannot be made using data from the TCCON or NOAA network sites without significant interpolation in space and time due to the geographically sparse sampling of the ground-based networks.

Figure 1 shows the latitude-time distributions of XCO_2_ obtained from NASA’s OCO-2 and JAXA’s GOSAT instruments^26,27^ and the differences with JAMSTEC’s atmospheric chemistry-transport model (ACTM) simulations for the period from September 2014 through October 2016 (up to May for GOSAT). Details on observational data selection, ACTM simulations and their processing are given in the Methods section. The OCO-2 minus ACTM results are shown for three combinations of terrestrial and oceanic CO_2_ fluxes, namely, CYC64 (Fig. 1b), IAV84 (Fig. 1c) and IAV84 + GFAS (Fig. 1d). The simulated XCO_2_ growth rates by ACTM_CYC64 and ACTM_IAV84 overestimated (typically by ~0.5 ppm) and underestimated (by up to 2.0 ppm), respectively, the observed growth rate over this 25-month period. The underestimation of ACTM_IAV84 develops most strongly during Sep-Nov 2015. The ACTM_IAV84 + GFAS simulation most closely follows the OCO-2 observations, compensating in particular for the underestimation after Nov 2015 (referred to as ‘best’ a priori for flux corrections). All ACTM simulations use the same emissions from FFC at the rate of ~10 PgC yr^−1^ (Table 1). However, the annual total land and ocean fluxes vary, e.g., −2.86, −6.24, and −4.77 PgC yr^−1^, respectively, for CYC64, IAV84 and IAV84 + GFAS cases for period July 2015 to June 2016. One striking difference for the April-July period is that GOSAT – ACTM differences (Fig. 1f,g,h) in the high northern latitudes (>30°N) are more negative than the OCO-2 - ACTM differences (Fig. 1b,c,d). This suggests a surface sofluxurce inversion would produce stronger sosinkurces in the northern high latitudes when GOSAT observations are used compared to using the OCO-2 observations.

**Table 1:** Global total CO_2_ fluxes used in the 3 ACTM simulations (column 2–6), and estimated flux corrections (column 7–10) for different time windows given in column 1 (Units: PgC). Note here that these values are not strictly mass balanced as the XCO_2_ differences are weighted by area of the 3 latitude bands, without knowing whether the mismatches at high latitudes in particular extend to the poles on either side.

Time window	A priori CO_2_ fluxes used for ACTM simulations					Patra *et al*.^#^ (2005b)	CO_2_ flux corrections from OCO-2 – ACTM differences^$^		
	FFC	CYC64	IAV84	IAV84 + GFAS	GFAS		CYC64	IAV84	IAV84 + GFAS
Oct 2014–Sep 2015	9.93	−2.86	−6.24	−4.27	1.97	2.67–2.73	−0.1–0.23	1.17–2.04	0.41–0.71
Oct 2015–Sep 2016	10.12	−2.86	−6.24	−5.57	0.67		−0.75–1.10	1.00–1.16	0.53–0.67
Jul 2015–Jun 2016 (main El Niño period)	10.08	−2.86	−6.24	−4.77	1.46		−0.18–0.29	1.50–2.18	0.77–1.09

^#^Range estimated from two different fits, with (Flux anomaly = 0.3539 + 1.4935 × MEI amplitude change) or without (=−1.0756 + 2.4579 × MEI amplitude change) the La Niña years.

^$^Range of estimation using two different approximations on area coverage (lower: latitudes covered by measurements; higher: global; refer to the main text for details).

**Figure 2:**
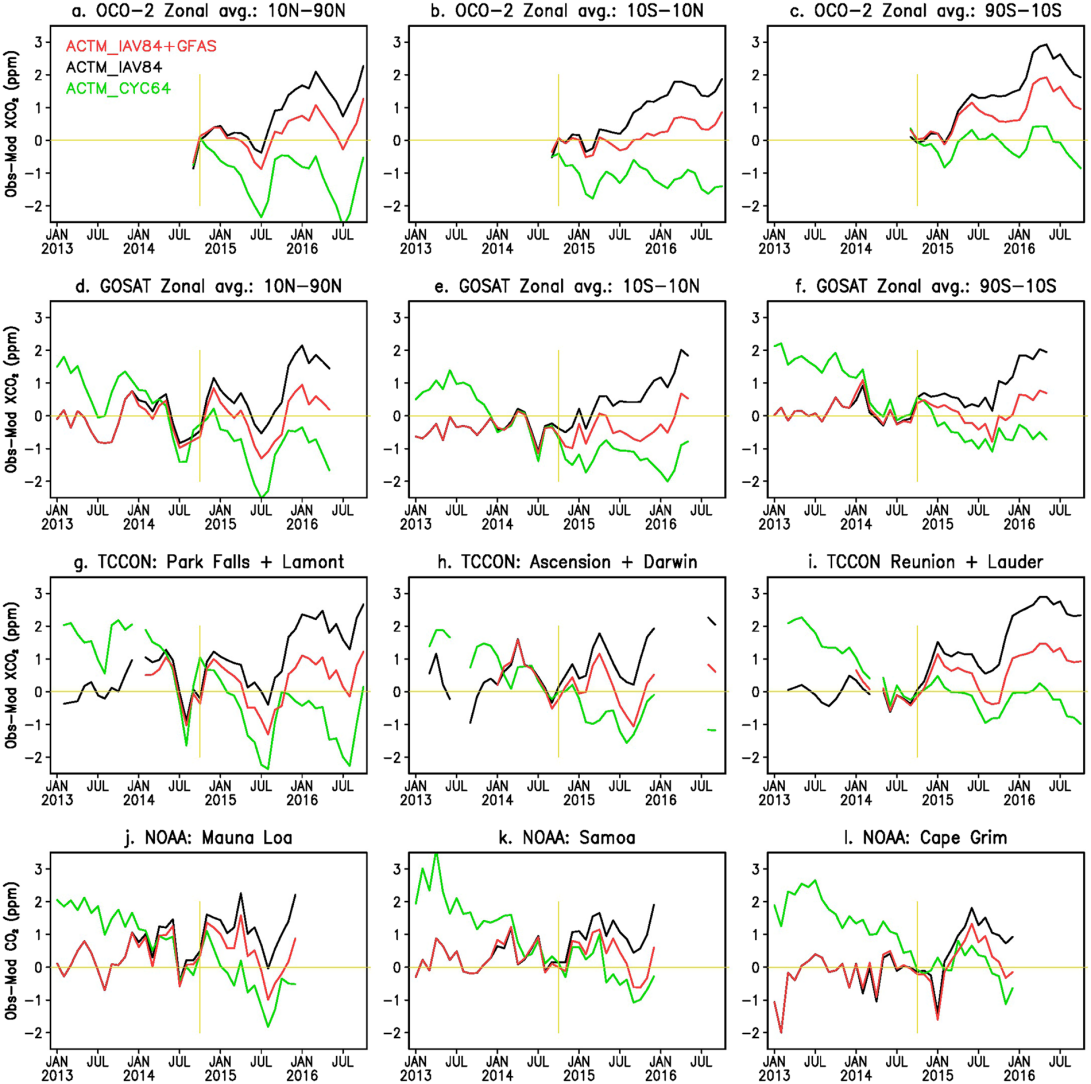
Observation-model comparisons of XCO_2_ and CO_2_ from different measurement systems. Time series of zonal mean differences in XCO_2_ (observation – model) for three broad latitude bands (top two rows). The differences in TCCON XCO_2_ and NOAA CO_2_ trends with ACTM simulations are shown in the bottom two rows. All three cases of model simulations (ACTM_CYC64: green, ACTM_IAV84: black, and ACTM_IAV84 + GFAS: red) are matched with observations on October 2014 (marked by vertical yellow line), which is chosen as the reference point for the calculation of XCO_2_ model-observation differences for calculating flux corrections. Note that the OCO-2 measurements are started from September 2014, GOSAT from 2009, TCCON from 2002, and MLO flask sampling from 1967. Common legends to all the subplots are given in top-left panel.

Figure 2a,b,c show comparisons of XCO_2_ as measured by OCO-2 and simulated by ACTM as zonal means for three broad latitude ranges for the period from September 2014 through October 2016. The latitude bands of 10°S–10°N (hereinafter referred to as tropics) and 10°–90° cover 88.6 and 210.7 million km^2^, respectively. When combined into 2.5° × 2.5° grid boxes, the OCO-2 data coverage for the latitude bands poleward of 10° varies from 30% to 50% of the total area. The region south of 10°S has the largest model–observation mismatches, with values up to 2 ppm, with major contributions from the American and Asian sectors, during April to August 2015. The ACTM_IAV84 simulation, on the other hand, most closely follows the OCO-2 observations until July 2015 for the region north of 10°N (Fig. 2a), suggesting that the FFC emissions are reasonably prescribed at an increase of 0.2 PgC yr^−1^ during 2014–2016 in the ACTM simulations and that the large model-observation mismatches at the later time are arising from the deficiencies in biospheric fluxes, both from land and ocean. The latest report of the Emissions Database for Global Atmospheric Research (EDGAR)^3^ suggest no increase in FFC emissions during 2014–2015 (no value for 2016 is yet available). Thus our estimation of biospheric emission during October 2014 to October 2016 could be underestimated by up to 0.2 PgC, which is assigned as FFC emission increase rate in our a priori model. The ACTM - OCO-2 differences show systematic decrease following the peak in February-March 2016, in particular for the southern latitudes, until October 2016, as the El Niño condition weakens (Fig. 2c).

Because the OCO-2 measurements started less than 6 months before the nominal onset of the 2014–2016 El Niño this data alone cannot be used for calculating anomalous CO_2_ emissions. We have used longer time record from GOSAT, TCCON (Total Carbon Column Observing Network)^28^ and NOAA cooperative global air sampling network^29^ measurements since January 2013 for defining the baseline. Here we report CO_2_ flux anomalies with respect to 2013–2014 as the aim of this study is to estimate anomalous CO_2_ release for the whole El Niño period. The ACTM_IAV84 simulation successfully simulated CO_2_ growth rate during January 2013 to September 2014 (seen as the differences around the 0-line) as measured by GOSAT (Fig. 2d,e,f), TCCON (Fig. 2g,h,i) and NOAA (Fig. 2j,k,l). For the October 2014 to October 2016 (El Niño) period, the ACTM_IAV84 + GFAS simulation most closely simulated the atmospheric XCO_2_ measured by GOSAT and TCCON, and also the NOAA flask observations (Fig. 2). Although the ACTM_IAV84 + GFAS simulation very well describes the time evolution of observed XCO_2_ in the tropics and most times for the region north of 10°N (mostly within 0.1 ppm), systematic underestimations of up to ~2.0 ppm are seen in the region south of 10°S by April 2016. The larger variability in model-observation mismatches in the northern latitude band (Fig. 2a,d,g) is probably an effect of strong terrestrial biospheric uptake and release cycle, which are not very well constrained by ACTM inversion system using *in situ* data only. This issue will be addressed later when flux corrections will be validated using TCCON observation.

### Global CO2 flux anomaly

Comparing the 3 ACTM simulations with OCO-2 and other measurements, we find that the global pyrogenic emission from GFAS of about 2.64 PgC, which in itself is subject to considerable uncertainties, is similar to our XCO_2_-based estimation for the 2015–2016 El Niño-induced extra carbon flux from vegetation fires, reduced net primary productivity, and errors in the assumed trends of FFC emissions during the period October 2014 – October 2016. Since the XCO_2_ values consist of vertically-integrated information for the whole atmospheric column, simple approximations can be applied for estimating CO_2_ flux corrections (in PgC month^−1^) from meridional atmospheric CO_2_ burden differences (PgC) at monthly time interval (see Methods). The estimated CO_2_ flux corrections are summarized in Table 1. For the ACTM_IAV84 + GFAS fluxes, the anomalous CO_2_ emissions aggregated over the ‘main El Niño period’ (defined by July 2015 to June 2016) are in the range of 2.23–2.55 PgC. Because the ACTM_IAV84 + GFAS simulation generally follows the observed OCO-2 XCO_2_ (Fig. 2a–c), we use this as the ‘best’ prior for CO_2_ flux correction. The best prior case introduces less error in the flux corrections as the transport of flux increments are ignored in our calculation method. The 0.32 PgC difference in emissions is due to extrapolation of XCO_2_ differences poleward in both hemispheres (Fig. 1d). The lower range of values in the 3 right columns are obtained without extending model-observation mismatches to the missing data grids. An effect of decay in El Niño condition since April 2016 is seen in reduction of CO_2_ flux anomaly for October 2015 – September 2016 (1.20–1.34 PgC), compared to October 2014 – September 2015 (2.38–2.68 PgC).

The range of estimated CO_2_ flux corrections is consistent with the empirical calculation of the CO_2_ flux anomaly (2.67–2.73 PgC) using its linear relationships with the MEI trend (Table 1)^15^. Using the CO_2_ flux anomaly and MEI trend relationship^15^, the CO_2_ flux anomaly for the 1997/1998 is estimated at 4.4–5.7 PgC, while that from the atmospheric-CO_2_ inversion was 6.7 PgC. A global CO_2_ emission anomaly of ~2 PgC is estimated for July 1997 – June 1998 due to fires alone^16^.

The annual mean CO_2_ residual land fluxes for the main El Niño period are then estimated as −3.15 (=−2.86–0.29), −4.06 (=−6.24 + 2.18) and −3.68 (−4.77 + 1.09) PgC yr^−1^ for the simulation cases ACTM_CYC64, ACTM_IAV84 and ACTM_IAV84 + GFAS for the control data screening. The July 2015 to June 2016 aggregated fluxes for ACTM_IAV84 + GFAS (best a priori) case are only weakly sensitive when OCO-2 data are screened for AMF < 3.5 and WL < 6 (−3.83 = −4.78 + 0.95 PgC) or AMF < 2.5 and WL < 6 (−3.75 = −4.78 + 1.03 PgC; ref. Table S1). The consistency over data screening and transport model cases provide us confidence on the adapted methodology for calculation of flux correction from model-observation XCO_2_ differences, and suggest that treatment of the data gaps do not significantly affect the estimation CO_2_ flux anomaly (2.48 ± 0.07 PgC; mean and 1-σ standard deviation based on 3 sensitivity cases for WL and AMF). The CO_2_ flux anomalies estimated from ACTM and GOSAT XCO_2_ differences is 2.65 (=1.70 for GFAS + 0.95 from XCO_2_ flux correction) PgC for the IAV84 + GFAS fluxes and period June 2015 to May 2016 (note one month difference with OCO-2) are also found to be in good agreement with those estimated using OCO-2.

**Figure 3:**
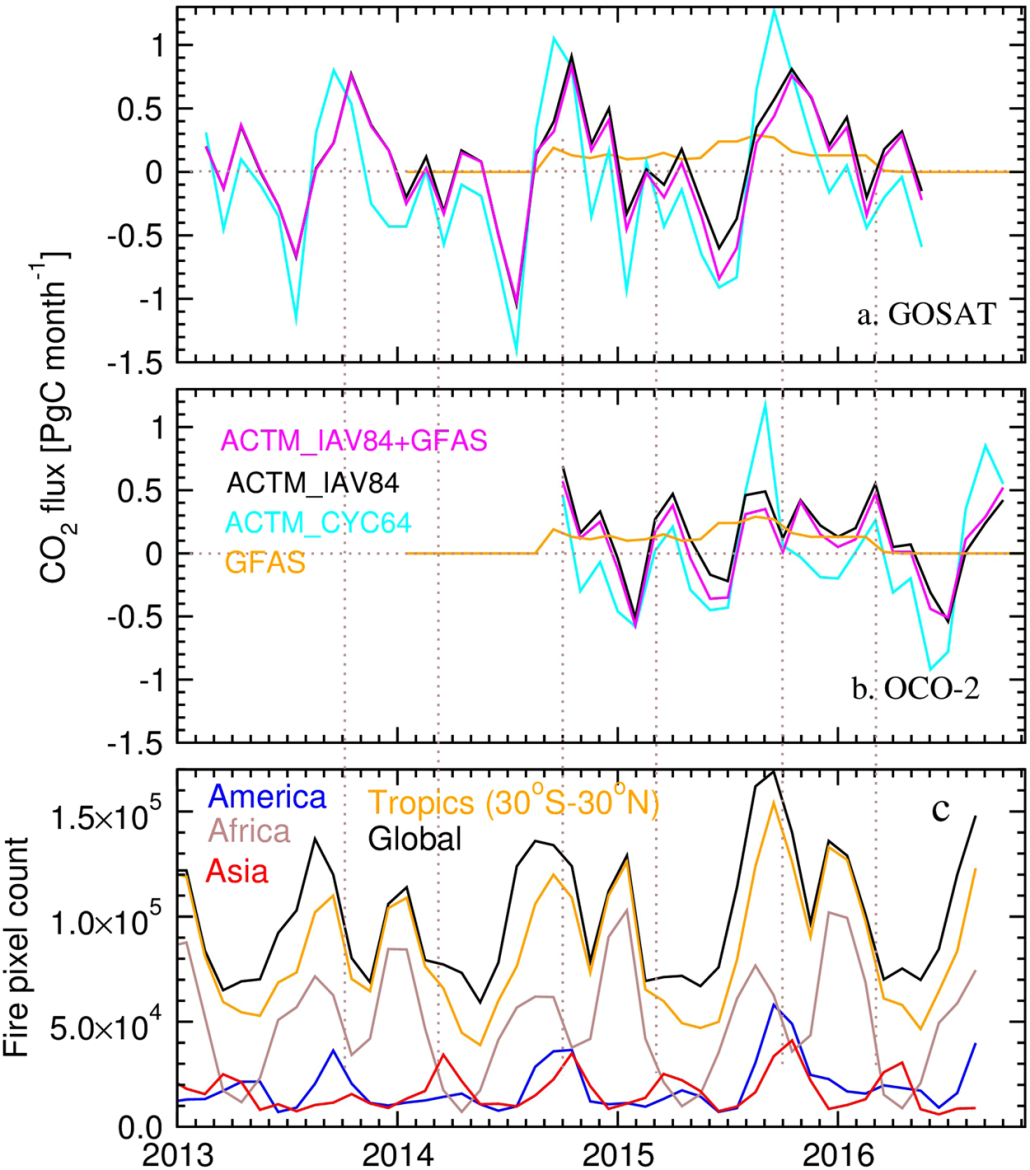
Global CO_2_ flux corrections and fire count variability. Global total CO_2_ flux corrections for the extended global latitudes, estimated from the GOSAT and ACTM (**a**; top), OCO-2 and ACTM (**b**; middle) differences and global total GFAS emissions, and fire-pixel counts for global, tropics (30°S–30°N) and by continental divisions for the tropics (**c**). Fire counts are taken from the Moderate-resolution Imaging Spectroradiometer (MODIS) Active Fire Products^30^ (ftp://fuoco.geog.umd.edu/modis/C5/cmg/monthly/hdf/).

Figure 3 shows the monthly variations in CO_2_ flux corrections along with the number of ~1 km^2^ pixels with fire, seen from the MODIS sensor onboard the Terra satellite^30^. The positive CO_2_ flux corrections for both GOSAT and OCO-2 show high coincidence with large fire counts, e.g., during September-October of 2014 and 2015, high CO_2_ emissions are caused by fires in maritime tropical Asia (mainly Indonesia) and America (mainly Brazil), and emissions during March-April 2015 can be linked to fires in the continental tropical Asia (Thailand and the neighboring countries)^14^. As seen from Fig. 3c, more than 90% of global fires (solid line) occur within the latitude band of 30°S-30°N (broken line), and are emitted as pulse in a one month time window. This result of anomalous XCO_2_ increase during the 2015–2016 El Niño can be assigned to CO_2_ emissions from the tropical land. Because the signal from the enhanced fires is correlated with drought, the CO_2_ observation based study cannot quantitatively discriminate the relative roles of reduction in biospheric uptake due to warmer and drier climate, and emissions from biomass burning. Interestingly, although the time-integrated GFAS emissions are in good agreement with tropical XCO_2_ increase, the timing of pulsed CO_2_ emissions during the fire events is not well represented. However, as a first guess, we estimate fire emissions to be ~0.76 PgC from the peaks in November 2015 and March 2016 (months following the large fire counts as marked by the dotted lines vertical lines in Fig. 3), which is 30–34% of the total flux anomaly for the main El Niño period.

### Meridional CO2 flux anomaly and flux validation using TCCON

**Figure 4:**
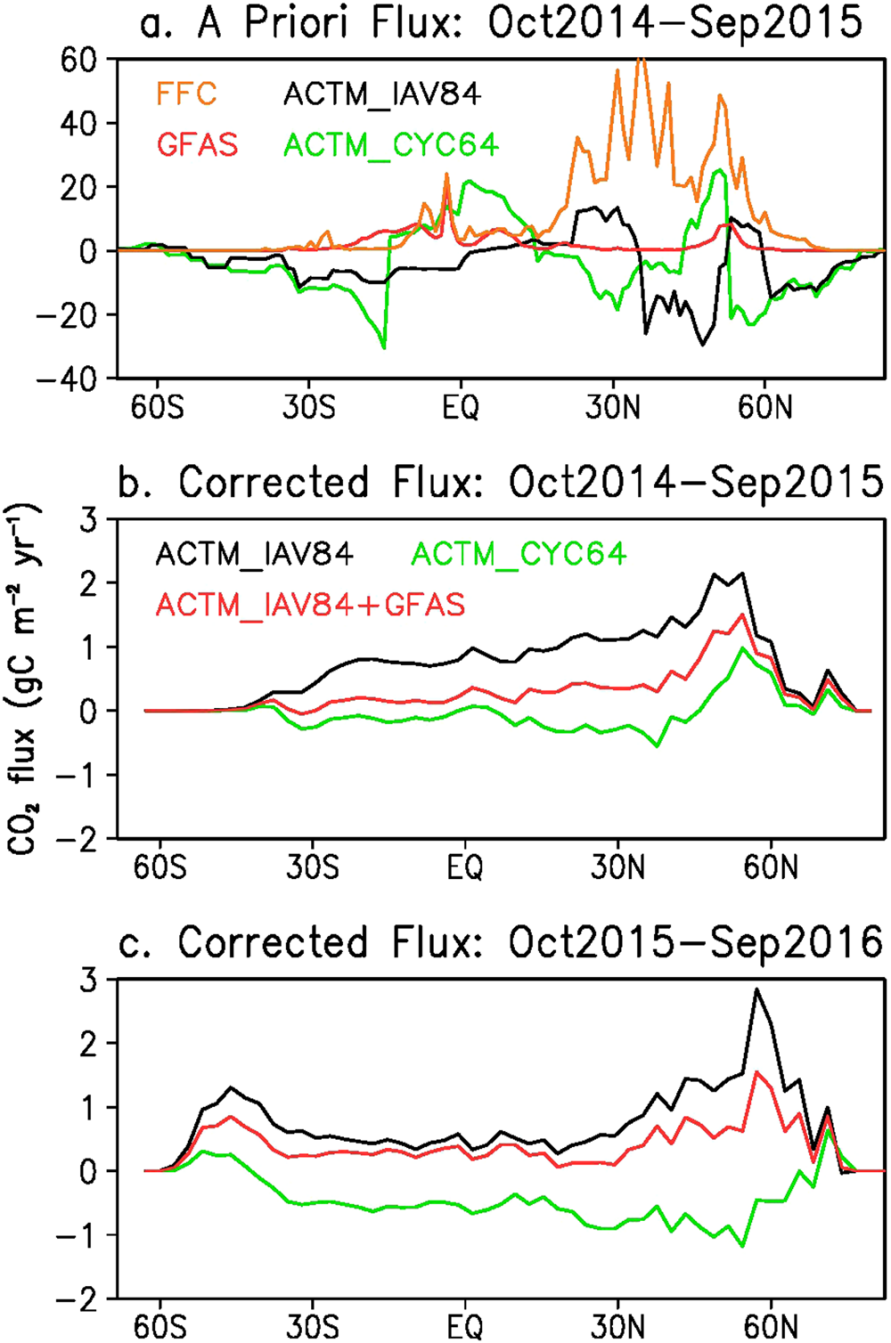
Meridional distributions of CO_2_ fluxes and flux corrections. (**a**) A priori fluxes for fossil-fuel and cement production, land and oceanic fluxes in ACTM_CYC64 and ACTM_IAV84, GFAS fire emissions averaged over October 2014 – September 2015. The flux corrections for the two separate years (averaged over: October – September) are shown for the 3 ACTM simulation cases (**b**,**c**; legends in **b** are common to both panels).

Figure 4 shows the meridional distributions of annual mean a priori fluxes and flux corrections using OCO-2 XCO_2_ observations. The flux corrections are found to be greatest at around 35–60°N (Fig. 4b,c), up to 10% of the rate of the total a priori biospheric (non-fossil) fluxes, which are of the order of ±20 gC m^−2^ yr^−1^ at these latitudes. In general, the flux corrections at all latitudes are smallest for the ACTM_CYC64 simulation and greatest for the ACTM_IAV84 simulation, but an overall source or a weak sink is observed during October 2014 – September 2015 (Fig. 4b). A clear sink tendency is developed for the period October 2015 – September 2016 for the ACTM_CYC64 case and slightly weaker source for the ACTM_IAV84 or ACTM_IAV84 + GFAS simulations (Fig. 4c). These suggest that the effect of El Niño on CO_2_ release from the biosphere has been moderated in the latter part of 2016 compared to that in 2015 (ref. also Table 1).

**Figure 5:**
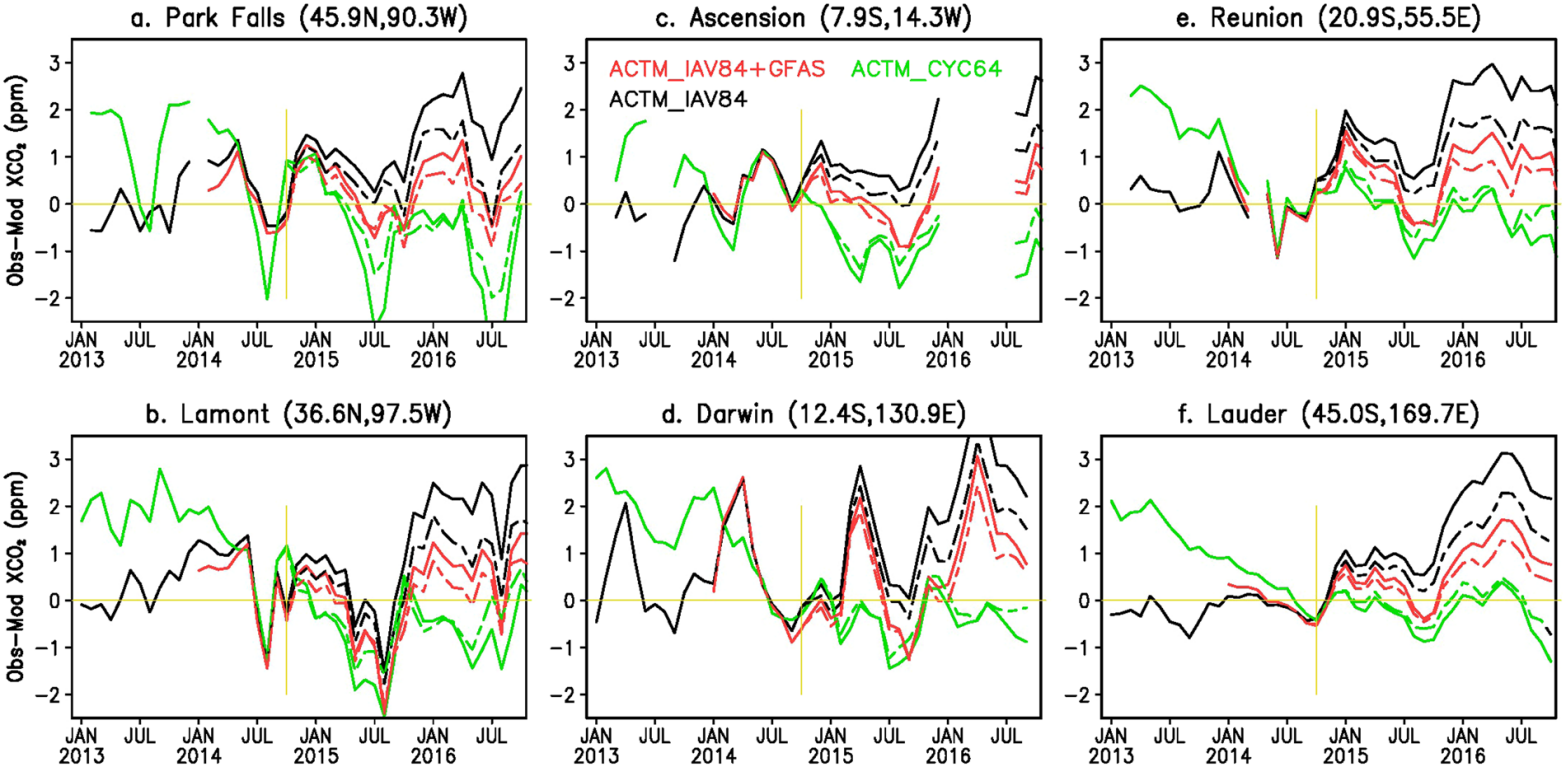
Comparisons of XCO_2_ as measured by TCCON and simulated by ACTM. The XCO2 time series are shown for 6 sites (as opposed to paired sites shown in Fig. 2g–i) for two sets of simulations, solid and broken lines are for ACTM runs using a priori and corrected fluxes, respectively. The statistics of TCCON-ACTM mismatches are given in Table S2, which are calculated from individual TCCON data.

Figure 5 shows the TCCON-ACTM mismatches for the simulations using a priori and corrected fluxes, calculated using individual XCO_2_ observations. We find that the best flux corrections are obtained for the best a priori case (ACTM_IAV84 + GFAS), where the root-mean-square (RMS) differences of TCCON-ACTM XCO_2_ are below 0.78 ppm for 5 out 6 sites (except for Darwin at 1.07 ppm). A reduction in RMS differences of 70–80% are found for this ACTM case. The simulation case of ACTM_CYC64 also achieved RMS differences close to 1.0 ppm or lower following the flux correction. However, the case of ACTM_IAV84 showed a mean RMS difference of 1.5 ppm after flux corrections are applied. Thus a good a priori ACTM simulation is critical for implementing this method of flux correction using OCO-2 measurements. One of the most encouraging improvement in ACTM – OCO-2 difference is seen for Park Fall. At this site, the differences were largest in July, which are reduced by half to ~1 ppm in 2015 and ~2 ppm in 2016 for the ACTM_CYC64 case (Fig. 5a), suggesting that the CO_2_ sinks should be increased in the northern mid-latitude region (green line in Fig. 4b,c). Such seasonal bias is not seen for ACTM_IAV84 case, but an overall reduction in sink in the northern mid-latitudes is suggested (consistent with Fig. 4b,c). Both the seasonal and annual biases are the lowest for the ACTM_IAV84 + GFAS case.

Following this validation, we conclude the CO_2_ flux anomaly to be 2.4 ± 0.2 PgC for the July 2015 – June 2016 period using the flux corrections obtained for ACTM_IAV84 + GFAS case only. An annual total land and ocean sink of 3.9 ± 0.2 PgC yr^−1^ during July 2015 – June 2016, for the assumed fossil fuel emissions of 10.1 PgC yr^−1^, contrasts the average sink of 6.2 PgC yr^−1^ during the reference year of 2014. This is in huge contrast to the July 1997 – June 1998 period, when the Earth’s surface acted as a net source of CO_2_ to the atmosphere. Since the atmospheric growth rate measured by the NOAA/ESRL at Mauna Loa is 3.05 ppm yr^−1^ for the main El Niño period, the global residual sink of 3.6 (=10.1–3.05*2.12) PgC yr^−1^ is fairly consistent with our results. The residual sink for 1998 based on Mauna Loa growth rate was 0.5 (=6.7–2.93*2.12) PgC yr^−1^.

**Figure 6:**
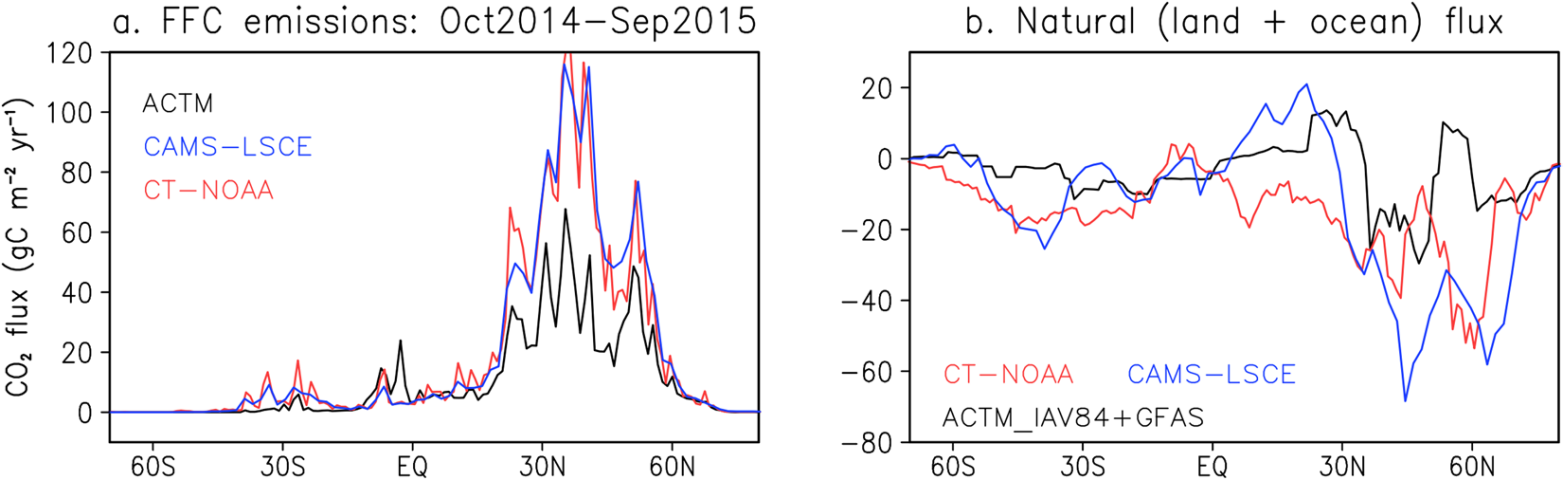
Comparison of a priori FFC CO_2_ emissions and total natural/biospheric (land + ocean) fluxes from inverse modelling. The fluxes from two independent traditional inversions are taken from CarbonTracker by NOAA (CT-NOAA; Peters *et al*., 2007; version: CT2016; www.esrl.noaa.gov/gmd/ccgg/carbontracker/) and Laboratoire des Sciences du Climat et de l’Environnement (LSCE) inversion results from CAMS (CAMS-LSCE; Chevallier *et al*.^32^; version: v15r4; http://apps.ecmwf.int/datasets/data/cams-ghg-inversions/).

In an attempt to gain further confidence in the ACTM corrected fluxes we compared the meridional gradients in CO_2_ fluxes from two other traditional inversions (Fig. 6). The traditional inversions are: CarbonTracker run from NOAA^31^ and Copernicus Atmosphere Monitoring Service (CAMS)^32^. The comparison suggests large differences between the inversion fluxes, and the differences showing strong dependence on a priori FFC CO_2_ emissions. Generally, the model assumed stronger FFC emissions also suggest stronger biospheric uptake, with particular distinctions in the northern mid-latitude region^33^. This leads us to conclude that the simple inversion system using XCO_2_ observations and ACTM simulations is usable for global CO_2_ flux anomaly calculation.

## Discussion

The powerful 2015–2016 El Niño has made a large impact on the Earth’s natural climate system, which in turn affected the terrestrial ecosystem. We analyzed the column-averaged CO_2_ dry mole fraction (XCO_2_) estimates from NASA’s OCO-2 observations collected between September 2014 and October 2016. We have also used the longer measurement records from JAXA’s GOSAT, TCCON ground-based XCO_2_ and NOAA *in situ* CO_2_ measurements in the analysis. Global simulations using JAMSTEC’s ACTM are performed for three combinations of terrestrial and oceanic CO_2_ fluxes: CYC64, IAV84 and IAV84 + GFAS, and a common field of emissions from fossil fuel consumption and cement production. The XCO_2_ and CO_2_ growth rates are slightly overestimated by ACTM_CYC64, but a greater underestimation was found for ACTM_IAV84 while compared with OCO-2 observations. The ACTM_IAV84 simulation successfully simulated CO_2_ growth rates during January 2013 to mid-2014. Thus the IAV84 + GFAS simulation produced the smallest model-data mismatch over the tropics when GFAS emissions were added from October 2014 (total emission of 2.64 PgC). We estimate that the El Niño event led to excess CO_2_ release to the atmosphere in the range of 2.23–2.55 PgC during July 2015 to June 2016, compared to the reference period of 2014. This CO_2_ release would be increased by 0.2 PgC if no increase in FFC emission was assumed.

In year 2015, about 0.76 PgC is emitted from fires, which is in the range of 30–34% of total CO_2_ flux anomaly. The OCO-2 based CO_2_ flux anomaly of 2015–2016 El Niño is comparable to that is estimated from an empirical relation of CO_2_ flux anomaly and ENSO index trends (2.67–2.73 PgC). Our estimated fire-induced CO_2_ flux anomalies disagree with those calculated from the GFED4.1 s total fire CO_2_ emissions of 1.64, 1.88 and 2.09 PgC for 2013, 2014 and 2015, respectively (anomaly ~0.2 PgC for 2015 relative 2014). and are more comparable to the 1997 and 1998 fire emission anomalies (~1 PgC) with global emissions of 2.75 and 2.67 PgC, respectively (http://www.falw.vu/~gwerf/GFED/GFED4/tables/GFED4.1s_CO2.txt)^14^.

The flux corrections based on OCO-2 measurements are validated using independent TCCON measurements, which suggest systematic reductions in TCCON-ACTM mismatches for the simulations using corrected fluxes compared to the a priori fluxes. A mean 1-σ standard deviation of 0.7 ppm is achieved for 6 TCCON sites for the period of October 2014 to October 2016 using the corrected fluxes. The flux correction method is applicable to satellite observations with near global coverage to calculate global CO_2_ flux anomalies at near real-time when a suitable a priori model simulation of atmospheric-CO_2_ is available, e.g., ACTM_IAV84 + GFAS case in this study. Based on our best a priori case, the global total flux anomaly is estimated to be 2.4 ± 0.2 PgC to the atmosphere as an effect of the El Niño, while the Earth’s surface acted as a net sink of CO_2_ by 3.9 ± 0.2 PgC during the period of July 2015 – June 2016.

## Methods

We used the bias corrected measurements of XCO_2_ from the ‘OCO-2 7 LITE LEVEL 2’ files^26^ (updated document at http://disc.sci.gsfc.nasa.gov/OCO-2/documentation/oco-2-v7; last accessed: 5 December 2016). These files only include those soundings that have passed the cloud screens and converged (xco2_quality_flag = 0). In addition, only those soundings that have a warn level (WL) less than 12 and air mass factor (AMF) less than 3.5 are used in this analysis (Control case), but no distinction is made for the different viewing modes of nadir, glint or target. All the data for the period extending from 06 September 2014 to 31 October 2016 are combined into 2.5° × 2.5° grid boxes at monthly time intervals for the convenience of analysis. Any grid containing less than 3 OCO-2 soundings (N) or an absolute model (ACTM_IAV84 + GFAS case) - observation XCO_2_ difference greater than 9 ppm is set to undefined. The limits for WL and AMF are chosen after testing different cut-off levels for making the gridded dataset. For example, use of AMF < 2.5 or < 3.5 did not produce large number of zonal-mean XCO_2_ differences greater than ± 1 ppm at most latitude bands (except at the high latitude edge of the satellite orbit) in all months. Similarly XCO_2_ differences greater than ±1 ppm were not found frequently for selection of WL < 6 or WL < 12. Various sensitivities of these data screening parameters are shown in the Supplementary Information (Fig. S1 and S1).

In addition, we have used selected measurements of XCO_2_ from the ground-based Total Carbon Column Observing Network (TCCON)^28^ and CO_2_ from the NOAA cooperative global air sampling network^29^ [Product: obspack_co2_1_CarbonTracker-NRT_v2.0_2016–02–12]. We have used the XCO_2_ data from TCCON sites at Lauder (45°S, 170°E)^34^, Reunion Is (21°S, 55°E)^35^, Darwin (12°S, 131°E)^36^, Ascension Is (8°S, 14°W)^37^, Lamont (37°N, 97°W)^38^ and Park Falls (46°N, 90°W)^39^. The *in situ* CO_2_ data are taken from Cape Grim (41°S, 145°E), Samoa (14°S, 171°W), Ascension Is (8°S, 14°W), Seychelles (5°S, 55°E), Barbados (13°N, 59°W), Mauna Loa (20°N, 156°W), Barrow (71°N, 157°W) and Alert (82°N, 62°W).

The four-dimensional (4D) distribution of CO_2_ mole fractions are simulated using the Center for Climate System Research/National Institute for Environmental Studies/Frontier Research Center for Global Change (CCSR/NIES/FRCGC) atmospheric general circulation model (AGCM)-based CTM (i.e., JAMSTEC’s ACTM)^40^. ACTM is run at a horizontal resolution of T106 spectral truncations (~1.125 × 1.125°), and 32 sigma-pressure vertical levels, and meteorology is nudged to horizontal winds and temperature from the Japanese 55-year Reanalysis (JRA-55)^41^. The following CO_2_ flux tracers are simulated by ACTM with an aim to encompass the observed CO_2_ growth rates during October 2014 to February 2016 (Table 1):

- *Flux CYC64*: This simulation is performed using the inverted land and ocean fluxes for the year 2008 from 64 land and ocean regions^40^. The global total flux for this inversion is −2.86 PgC yr^−1^ (Table 1), relatively weaker sink and thus over-predict the atmospheric CO_2_ growth rate for the decade of 2010s.
- *Flux IAV84:* Monthly-mean CO_2_ fluxes for 84 land and ocean regions corresponding to year 2011 are taken from an 84-region inverse model^42^. The global total flux for this inversion is −6.24 PgC yr^−1^, relatively stronger sink and thus under-predict the atmospheric CO_2_ growth rate for the decade of 2010s.
- *Flux GFAS:* The fire-related daily CO_2_ emissions are taken from the Global Fire Assimilation System (GFAS; version 1.2)^19^. The GFAS emissions are added to IAV84 fluxes from October 2014 onwards, and is used here as a proxy for anomalous CO_2_ emission, not specifically as a quantification of fire emission. Since more than 90% of GFAS emissions occur in the 20°S-20°N, this is regarded as a surrogate for tropical land flux anomaly.

Interannually varying a priori emissions for fossil fuel consumption and cement production (*FFC*) are taken from the Emissions Database for Global Atmospheric Research (EDGAR, v4.2)^3^. Same for all 3 cases. The spatial distribution of emissions for 2010 is repeated for all the later years with a 0.2 PgC yr^−1^ increase globally. This assumption of emission increase rate has identical, but compensating, effects on the estimation of interannual variations in CO_2_ fluxes.

The CO_2_ flux tracer simulations are started on 01 January 2005. We then combine the CO_2_ flux tracers to get 4D CO_2_ concentrations, as ACTM_CYC64 (=FFC + CYC64), ACTM_IAV84 (=FFC + IAV84), ACTM_IAV84 + GFAS (=FFC + IAV84 + GFAS). These 3 combinations of model CO_2_ concentrations allow us to cover the whole range of XCO_2_ increase observed by OCO-2 and TCCON, and CO_2_ at NOAA sites. The model CO_2_ values are adjusted by −1.80, −1.45 and −1.45 ppm, respectively, for ACTM_CYC64, ACTM_IAV84 and ACTM_IAV84 + GFAS on 01 September 2014, coinciding with the start of data collection by OCO-2. This adjustment leads to no flux correction for September 2014. The vertical profiles of CO_2_ are first sampled at the location and time of individual OCO-2 measurements, and then convolved with the a priori profiles and averaging kernels of OCO-2, GOSAT and TCCON for calculating ACTM XCO_2_ values^43^.

Note that the ACTM_IAV84 simulation successfully simulated the CO_2_ concentrations for the time evolution and tropospheric profiles over Asia for the period 2007–2012^41^. Also shown here that the CO_2_ growth rates are well simulated by ACTM_IAV84 at the selected TCCON and NOAA ground-based measurement sites for January 2013 to mid-2014. Thus any differences in time evolution during the period September 2014 to February 2016 of OCO-2 data analysis can be attributed to excess CO_2_ releases associated with the El Niño event, relative to the 2014 mean.

Model XCO2 are calculated^43^ by convoluting model CO2 profile (CO_2_^ACTM^) with that of the a priori profile (CO_2_^pri^°^r^) and column averaging kernels (A_i_) of instrumental sensitivity to different layers of the atmosphere (P_i_, i = 20, 20 and 71 for OCO-2, GOSAT and TCCON, respectively).

1 $$\begin{array}{rcl}XC{O}_{{2}}^{ACTM} & = & {\sum }_{i}\,(C{O}_{{2}\,\quad \quad i\cdot }^{prior}.d{P}_{i})+{\sum }_{i}{A}_{i}({\sum }_{i}C{O}_{{2}\,\quad \quad i\cdot }^{ACTM}.d{P}_{i}-{\sum }_{i}C{O}_{{2}\,\quad \quad i\cdot }^{prior}d{P}_{i})/\\  &  & ({\sum }_{i}d{P}_{i}/cH2{O}_{i})\end{array}$$

dP_i_ is the thickness of each pressure layers. Water vapour corrections are applied to both the model and all TCCON column observations as are reported in dry air mole fractions. The correction term for each altitude level (*i*) is defined as:

2 $$cH{2}O=g.{M}_{air}({1}.{0}+{q}^{dry}.{M}_{H{2}O}/{M}_{air})$$

where, q^dry^ = q/(1−q) and q is specific humidity (mass fraction, kg/kg). M_H2O_ = 18.02 and M_air_ = 28.964 g/mole. Gravity ‘g’ is corrected for altitude (refer for further details: https://tccon-wiki.caltech.edu/Network_Policy/Data_Use_Policy/Auxiliary_Data).

Since the XCO_2_ values consist of vertically-integrated information for the whole atmospheric column, assuming that the simulated carbon atmospheric fluxes are perfect, simple approximations can be applied for estimating CO_2_ flux corrections (in PgC month^−1^) from sub-hemispheric atmospheric CO_2_ burden differences (PgC) at monthly time interval.

3 $$Burden\,difference=\Sigma (XC{O}_{{2}}\,difference\times area\,of\,the\,grid\times air\,density)$$

4 $$C{O}_{{2}}\,flux\,correction=d(Burden\,difference)/dt$$

where the XCO_2_ difference is the observed minus model values, area of the grid is latitude dependent and air density is calculated as the air mass overhead each 2.5 × 2.5 grid from ACTM air density. The difference in the burden mismatches between October and September 2014 is assigned to the flux correction for October 2014. For these flux estimations in the control case, missing areas are filled by the mean values of the observed – model differences for the 3 latitude bands. This is done based on an assumption that the mean differences will be transported within the semi-hemispheric regions within months by the rapid zonal mixing. In this simple method, we do not expect to resolve the evolution of flux corrections at less than a 1-month time resolution or the contrast between the continents and between land-ocean. However, this method is applicable for near real-time monitoring of biospheric health of Earth’s ecosystem without significant additional investment.

This method of flux corrections is valid only for sub-hemispheric scales since the zonal transport circulates air masses several times around each of the 3 broad, zonal bands within one month. This method suffers from the extrapolation of data to the missing observation grid boxes. For example, OCO-2 soundings covered a maximum of 70, 70 and 60% of the 2.5 × 2.5° grid cells in the latitudes bands of 90°S-10°S, 10°S-10°N, 10°N−90°N, respectively. In the latitude bands poleward of 10°, monthly data coverage can be as low as 30% in the winter hemisphere. Data coverage in the tropical latitudes suffers mainly from cloud cover (in addition to the model transport error), sometimes for longer than a month, and are approximated at modelers discretion by choosing not to modify the priors or applying a time correlation. The fraction of missing data area will increase further when analyzed for smaller than 2.5° × 2.5° grid sizes. Note that this method cannot be employed for the *in situ* measurement network without significant extrapolation in space and for the fact that the ground measurement sites do not cover the majority of the continental source regions^44^.

As opposed to the site-based data analysis^12,13,15^ for CO_2_ flux anomaly, this method based on differences between the observation-model difference does not require a long time series of data. As shown here, only one year of reference is sufficient, (2014 used in this analysis). Another major advantage of this analysis comes from the near uniform data coverage over the continents of tropical Asia, Australia, South America and Africa, which are very sparsely observed by the *in situ* measurement networks, providing a true global CO_2_ flux signal. The traditional analyses mentioned earlier in the Introduction focused on one site, which is often under the influence of regional or local flux signals.

Finally, we are also able to validate the flux corrections from ACTM – OCO-2 XCO2 differences using an independent set of TCCON observations. The zonal mean flux corrections (Fig. 4) are simulated using ACTM and XCO2 signals added to their respective a priori simulations. The results are presented in Fig. 5, which show clear reduction in ACTM – OCO-2 differences after the corrected flux simulations (Table S2). Flux corrections using ACTM and OCO-2 XCO_2_ are also compared with CarbonTracker and CAMS traditional inversion results showing greater influence of fossil fuel a priori emissions on the estimated biospheric flux compared to the differences arising from flux estimation methods (Fig. 6).

## Electronic supplementary material

Supplementary information
