# Supplementary material for: The Orbiting Carbon Observatory (OCO-2) tracks 2–3 peta-gram increase in carbon release to the atmosphere during the 2014–2016 El Niño
Source: Sci Rep. 2017 Oct 19;7:13567. doi: 10.1038/s41598-017-13459-0 (PMC5648889; doi:10.1038/s41598-017-13459-0)
Supplement: Supplementary file 1 — Supplementary information [file 41598_2017_13459_MOESM1_ESM.pdf]

Supplementary information:

The Orbiting Carbon Observatory (OCO-2) tracks 2-3 peta-gram increase in carbon release to the atmosphere during the 2014-2016 El Niño

Prabir K. Patra[1,*], David Crisp[2], Johannes W. Kaiser[3], Debra Wunch[4], Tazu Saeki[1], Kazuhito Ichii[1], Takashi Sekiya[1], Paul O. Wennberg[5], Dietrich G. Feist[6], David F. Pollard[7], David W. T. Griffith[8], Voltaire A. Velazco[8], M. De Maziere[9], Mahesh K. Sha[9], Coleen Roehl[5], Abhishek Chatterjee[10], Kentaro Ishijima[11]

Time window	A priori CO_2 fluxes used for ACTM simulations					Patra et al. [#] (2005b)	CO_2 flux corrections from OCO-2 – ACTM differences[$]		
	FFC	CYC64	IAV84	IAV84 +GFAS	GFAS		CYC64	IAV84	IAV84 +GFAS
Oct 2014 - Sep 2015	9.93	–2.86	–6.24	–4.27	1.97		–0.21 - –0.49	0.74 - 1.80	0.19 - 0.49
Oct 2015 - Sep 2016	10.12	–2.86	–6.24	–5.57	0.67		–0.58 - –1.31	0.38 - 0.77	0.17 - 0.33
Jul 2015 - Jun 2016 (main El Niño period)	10.08	–2.86	–6.24	–4.77	1.46	2.67 - 2.73	0.03 - –0.14	0.67 - 2.04	0.36 - 1.03

Table S1: Global total CO_2 fluxes as in Table 1, but for a sensitivity case when the OCO-2 data are screened as WL<6 and AMF<2.5. Note that the numbers only in the 3 columns on the right are changed.

Sites	ACTM using a priori fluxes			ACTM using corrected fluxes		
	CYC64	IAV84	IAV84+GFAS	CYC64	IAV84	IAV84+GFAS
Park Falls	1.71	1.98	0.71	1.16	1.30	0.52
Lamont	1.39	2.07	0.98	1.03	1.36	0.78
Ascesion Is	1.54	1.78	0.61	1.19	1.19	0.49
Darwin	1.02	2.62	1.34	0.79	1.95	1.07
Reunion Is	0.92	2.44	0.98	0.64	1.72	0.70
Lauder	0.80	2.29	0.92	0.58	1.66	0.65

Table S2: TCCON – ACTM mismatches for the simulations using a priori and corrected fluxes are given as 1-σ standard deviations. Note that the mismatches are always lower for the a ACTM simulations using corrected flux (3 right-most columns).

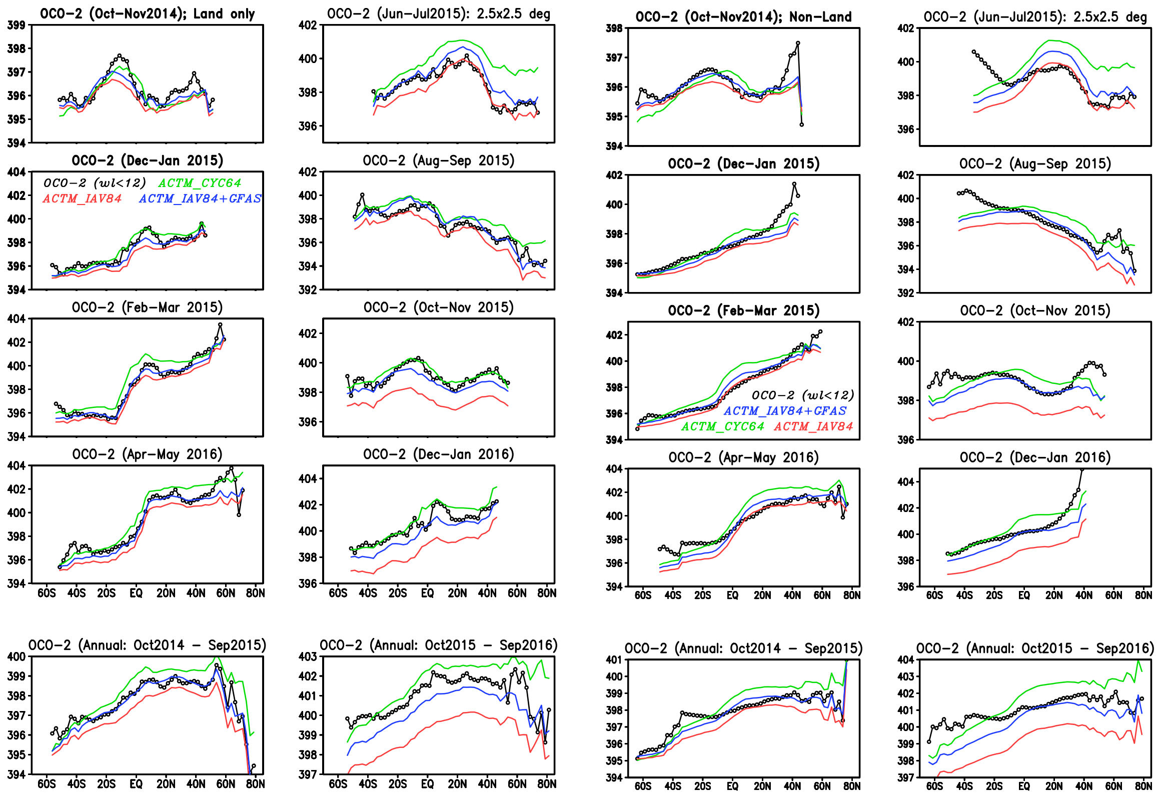

Figure S1: Latitudinal profiles of XCO2 as observed by OCO-2 and simulated by ACTM over the land regions (two columns on the left) and ocean regions (two columns on the right). Top 4 rows are for two monthly averages during October 2014 – January 2016, and the bottom row is for annual mean for the periods October 2014 – September 2015 and October 2015 – September 2016.

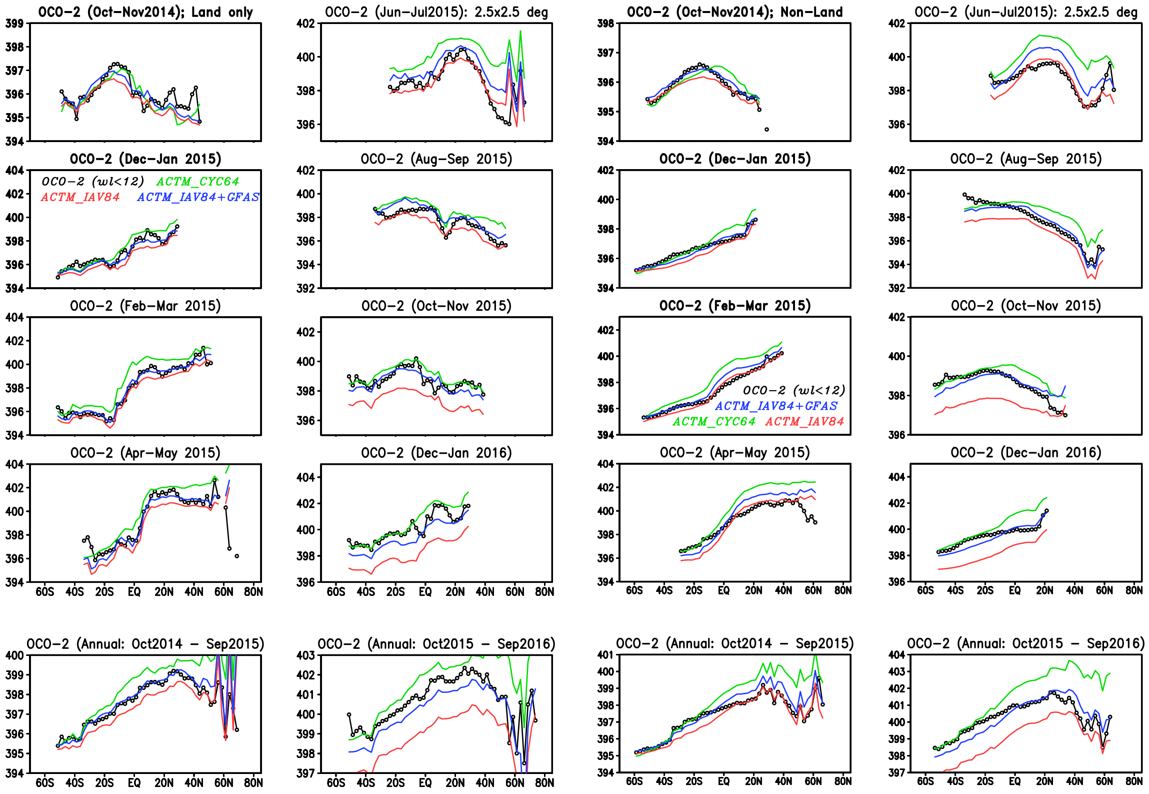

Figure S2: Latitudinal profiles of XCO2 as observed by OCO-2 and simulated by ACTM as in Figure S1, but when OCO-2 data are screened for WL<6 and AMF<2.5. This stricter screening reduced the data coverage poleward in both the hemispheres, and thus some of the extreme OCO-2 and ACTM mismatches (say, for Jun-July 2015 in the southern hemisphere over ocean), or the shape of the meridional gradients (say, for Oct-Nov 2014 over land)
